# Impact of obstructive sleep apnea on gut microbiome of patients with symptomatic intracranial atherosclerotic stenosis

**DOI:** 10.3389/fnagi.2026.1713733

**Published:** 2026-01-22

**Authors:** Shuoxi Liao, Hui Yang, Li Song, Lingli Shi, Zhike Lan, Wenrong Zhao, Zeyan Bao, Qiongli Hu, Xiaomei Tang, Sidian Zhuang, Huidi Wang, Shuisheng Zhong

**Affiliations:** 1 Department of Neurology, Guangdong Sanjiu Brain Hospital, Medical College of Jinan University, Guangzhou, Guangdong, China; 2 Microbiome Medicine Center, Department of Laboratory Medicine, Zhujiang Hospital, Southern Medical University, Guangzhou, Guangdong, China

**Keywords:** dysbiosis, gut microbiome, metabolic function, obstructive sleep apnea syndrome, symptomatic intracranial atherosclerotic stenosis

## Abstract

**Introduction:**

Obstructive sleep apnea syndrome (OSAS) is positively associated with increased risks of ischemic stroke. Patients with stroke exhibit remarkable gut microbiota dysbiosis. However, the impact of OSAS on gut microbiota of patients with symptomatic intracranial atherosclerotic stenosis (sICAS), one of the most common causes of stroke, remains unknown.

**Methods:**

This study included patients with sICAS, the severity of OSAS was defined by the apnea-hypopnea index (AHI). AHI < 5 was considered no sleep apnea, AHI 5–15 was defined as mild OSAS, AHI 15–30 as moderate OSAS, and AHI > 30 as severe OSAS. Fecal samples were collected and subjected to 16 s rRNA gene sequencing. PICRUSt2 was used to predict the functional properties of the bacterial communities.

**Results:**

In total, 99 sICAS patients were included, with No-OSAS (*N* = 22), Mild (*N* = 25), Moderate (*N* = 30), and Severe (*N* = 22). Patients with OSAS exhibited significantly altered gut microbiota composition compared to those without sleep apnea, characterized by increased abundances of pathogens such as *Escherichia-Shigella* and decreased abundances of beneficial microbes such as short-chain fatty acids-producing bacteria *Blautia*. Importantly, these microbes were significantly associated with AHI. Several microbial metabolic pathways such as Peptidoglycan biosynthesis, C5-branched dibasic acid metabolism, and Pantothenate and CoA biosynthesis were downregulated with OSAS.

**Conclusion:**

OSAS is associated with gut dysbiosis and altered microbial metabolic functions in patients with sICAS.

## 1 Introduction

Obstructive sleep apnea (OSA), also referred to as obstructive sleep apnea syndrome (OSAS), is a sleep-related breathing disorder characterized by recurrent episodes of partial (hypopnea) or complete (apnea) upper-airway collapse during sleep, resulting in intermittent hypoxemia and sleep fragmentation (Platon et al., 2023). Clinically, OSAS commonly presents with loud snoring, witnessed apneas, and excessive daytime sleepiness. OSAS is highly prevalent and underdiagnosed worldwide, a large modeling study estimated that approximately 936 million adults aged 30–69 years have at least mild OSA (AHI ≥ 5 events/h) and approximately 425 million have moderate-to-severe OSA (AHI ≥ 15 events/h), for which treatment is generally recommended (Benjafield et al., 2019). Prevalence increases with age and adiposity, making OSA particularly common in older populations (Crowley, 2011). OSAS substantially impairs health-related quality of life through non-restorative sleep and daytime dysfunction, including sleepiness/fatigue, reduced physical and social functioning, mood symptoms, and increased risk of drowsy driving and accidents (Huang et al., 2016).

Epidemiological surveys have revealed a strong association between the occurrence of OSAS and the increased risk of metabolic diseases (Kim et al., 2021), cardiovascular diseases (Gottlieb, 2021; Yeghiazarians et al., 2021), and cerebrovascular diseases (Baillieul et al., 2022). Stroke is the leading cause of adult disability and the second leading cause of death globally (GBD 2016 Stroke Collaborators, 2019). Intracranial atherosclerotic stenosis (ICAS) refers to atherosclerotic narrowing of the major arteries within the skull (e.g., intracranial internal carotid, middle cerebral, vertebral, and basilar arteries), which can compromise cerebral perfusion and precipitate ischemic events (Chen et al., 2024). ICAS is a leading cause of ischemic stroke worldwide and shows marked ethnic and geographic disparities, accounting for approximately 10% of ischemic strokes in the United States but a substantially higher proportion in Asian populations (often reported at approximately 30 to 50% in stroke cohorts) (Feng et al., 2019; Yaghi et al., 2019). Symptomatic ICAS (sICAS) denotes stenosis associated with a recent ischemic stroke or transient ischemic attack attributable to the affected intracranial artery, and it carries a high risk of recurrent stroke, particularly in severe stenosis (Chen et al., 2024). ICAS is one of most common causes of stroke, representing the highest recurrence rate in comparison to other etiologies (Wang et al., 2014). Although stroke and OSAS are intertwined conditions, sharing many common risk factors and comorbidities (Chen and Chen, 2021), researchers have suggested OSAS to be a pre-existing condition of stroke (Baillieul et al., 2022; Ott et al., 2020), which means OSAS is a potential cause of stroke. However, the underlying mechanisms remain incompletely understood.

From an etiological and epidemiological perspective, OSAS and ICAS converge in aging populations. Both conditions become more common with advancing age and frequently cluster with cardiometabolic risk factors such as obesity, hypertension, diabetes, and dyslipidemia (Benjafield et al., 2019). Beyond shared risk profiles, OSAS-related intermittent hypoxemia and sleep fragmentation can trigger sympathetic activation, oxidative stress, systemic inflammation, and endothelial dysfunction, these are pathways that are implicated in atherosclerosis development and progression (Chen et al., 2021). Consistently, observational evidence suggests that moderate-to-severe OSAS is associated with the presence of ICAS (Song et al., 2017). Therefore, clarifying biological intermediates that may link OSAS to vascular disease in patients with sICAS is clinically important.

Over the past decade, the gut microbiota has emerged to be a crucial contributor to the etiology of stroke (Honarpisheh et al., 2022; Björkegren and Lusis, 2022). Our previous work and studies of others have reported a dysbiotic gut microbiota structure in patients with ischemic stroke (Yin et al., 2015; Bao et al., 2024; Benakis et al., 2016). Metabolites derived from the gut microbiota are important mediators in the development of atherosclerosis. Trimethylamine N-oxide (TMAO), whose production requires gut microbial enzyme modifications, has been widely recognized as a prothrombotic metabolite by increasing platelet reactivity and vascular inflammation (Tang et al., 2013; Canyelles et al., 2023). Gut microbiota-derived lipopolysaccharide (LPS), which can induce endothelial dysfunction and oxidative stress, also contributes to atherosclerosis by promoting chronic inflammation (Wang et al., 2022). On the other hand, short-chain fatty acids (SCFAs), which are produced via degradation of carbohydrates from food by the gut microbiota, exhibit anti-inflammatory effects by inhibiting histone deacetylase (HDAC) activity and decreasing pro-inflammatory cytokine production (Li et al., 2018). Thus, the gut microbiota represents a critical contributor to the pathogenesis of atherosclerosis. However, whether the occurrence or severity of OSAS participate in the changes in gut microbiota in patients with symptomatic intracranial atherosclerotic stenosis (sICAS), remains largely elusive.

We therefore aimed to explore whether the gut microbiota was changed in sICAS patients complicated with OSAS, and investigate the gut microbiota profile in the subgroups divided by the severity of OSAS, and further investigate the functional differences in the microbial metabolic pathways between groups.

## 2 Methods

This study was conducted in the Department of Neurology, Guangdong Sanjiu Brain Hospital, Guangzhou, Guangdong, China from September 2021 to March 2024. The inclusion criteria of this study were as follows: (i) patients with an age >18 years; (ii) confirmed stroke owing to acute ischemic stroke [according to standardized clinical diagnosis with brain scan including either computed tomography (CT) or magnetic resonance imaging (MRI) and magnetic resonance angiography (MRA)]. And sICAS was confirmed by computed tomography angiography (CTA) or digital subtraction angiography (DSA). The exclusion criteria of this study were as follows: (i) patients with an explicit etiology of stroke not owing to sICAS (e.g., cardioembolism stroke, perivascular procedural stroke, cervical artery dissection); (ii) patients complicated with severe comorbid diseases or medical condition (e.g., respiratory failure, congestive cardiac failure, renal failure, hepatic failure or severe liver dysfunction); (iii) patients with a history of consuming prebiotics, probiotics or antibiotics within 1 month before admission.

### 2.1 Ethics approval and informed consent

This study involving human participants was reviewed and approved by the Ethics Committee of Guangdong Sanjiu Brain Hospital (approval no. 2021-01-024). The study was conducted in accordance with the Declaration of Helsinki. All participants provided written informed consent prior to recruitment.

### 2.2 Assessment of OSAS

The severity of OSAS was determined by the results of a polysomnography (PSG) examination. The participants were divided into two groups based on the apnea hypopnea index (AHI), with those with AHI < 5 considered no sleep apnea (NO.OSAS group) and those with AHI ≥ 5 considered sleep apnea (OSAS group). The participants were further divided into 4 subgroups based on the AHI, AHI < 5 was considered no sleep apnea, AHI 5–15 was defined as mild OSAS, AHI 15–30 as moderate OSAS, and AHI > 30 as severe OSAS.

### 2.3 Clinical data and fecal sample collection

The clinical data and medical history of patients were collected by trained neurologists from Guangdong Sanjiu Brain Hospital. Fresh fecal samples were obtained from each participant within 48 h after admission. Samples were collected into pre-labeled sterile, DNA-free 2 mL tubes (ThermoFisher, Cat No. 508-GRD-Q) using a disposable sterile spatula. No preservative or stabilizing reagent was added. After collection, the tubes were immediately placed in a sealed biohazard bag and kept on ice during transport to the laboratory within 3 h. Upon receipt, each sample was briefly homogenized and aliquoted (approximately 500 mg per aliquot) to minimize repeated freeze–thaw cycles, and all aliquots were stored at −80 °C until DNA extraction.

### 2.4 DNA extraction and PCR

Fecal genomic DNA was extracted using the ALFA-SEQ Stool DNA Kit (FINDROP, Guangzhou, China; Cat No. DZ309-03) based on silica spin-column purification. Briefly, 0.5 g fecal material was added to a zirconium bead beating tube, followed by 750 μL LA and 60 μL LB (added sequentially; LA and LB were not premixed), and vortexed to homogenize. The tube was incubated in a 90 °C water bath for 15 min, then bead-beaten at 3200 rpm for 30 s with a 30 s pause, repeated for two cycles, followed by centrifugation at 12,000 rpm for 3 min. The supernatant was transferred to a new tube and mixed with 250 μL R1 and 200 μL R2, centrifuged again (12,000 rpm, 3 min), and the clarified supernatant was combined with an equal volume of B4. The mixture was loaded onto the gDNA spin column (≤700 μL per loading), centrifuged at 12,000 rpm for 1 min, and the loading step was repeated until all lysate passed through the column. The column was washed with 600 μL wB1 and 600 μL wD1 (ethanol-diluted (Macklin, Cat No. E809061) working solutions), followed by a 2 min dry spin (12,000 rpm). DNA was eluted with 50–100 μL EB preheated to 65 °C, incubated for 2 min at room temperature, and centrifuged at 12,000 rpm for 1 min. The extracted DNA was stored at −20 °C until downstream analyses.

The amplification PCR was performed with genomic DNA as a template, targeting specific sequencing regions using the 16S V4 Region Primers (515F: GTGCCAGCMGCCGCGGTAA and 806R: GGACTACHVGGGTWTCTAAT), employing a BioRad S1000 PCR system (Bio-Rad Laboratory, CA). To verify the fragment size and concentration, the PCR products were analyzed via electrophoresis on a 1.5% agarose gel. The concentration of the PCR products was then measured using GeneTools Analysis Software (Version 4.03.05.0, SynGene), and the necessary volume for each sample was calculated based on equal mass principles. The PCR products were subsequently combined and purified with the E. Z. N. A. Gel Extraction Kit (Omega, USA; Cat No. D2500-02), with the target DNA fragments being eluted in TE buffer.

### 2.5 16 s rRNA sequencing

The library was constructed following the standard protocol of the ALFA-SEQ DNA Library Prep Kit (FINDROP, Guangzhou, China; Cat No. NDM001E-03), and the fragment size distribution was examined using the Qsep400 High-Throughput Nucleic Acid & Protein Analysis System (Hangzhou Houze Biotechnology, China). The library concentration was quantified with a Qubit 4.0 fluorometer (Thermo Fisher Scientific, Waltham, USA). Amplicon libraries were then subjected to PE250 sequencing on the Illumina iSeq 100 platform. Raw sequence data were processed and subjected to quality control using QIIME2 with default settings. The script pick_closed_reference_otus.py in QIIME was utilized for OTU clustering based on references, with USEARCH61 operating in reference mode to search the Greengenes database (version 13_8) and create the BIOM file. At a 97% similarity threshold, sequences were clustered into OTUs. The default mode of the Greengenes database was used to determine the phylogenetic relationships and taxonomic classifications of the representative OTU sequences.

### 2.6 Statistical analysis

The statistical analysis was performed with IBM SPSS Statistics version 24.0. Baseline characteristics of patients were described using mean ± standard deviation for continuous variables and counts (percentages) for categorical variables, depending on the type of data. Differences in baseline characteristics were assessed using either an unpaired Student’s *t*-test or a Chi-square test, as appropriate. Alpha diversity was evaluated using the Chao1, Shannon, and Simpson indices. Differences in alpha diversity were tested with either an unpaired Student’s *t*-test or a Kruskal–Wallis test, followed by Dunn’s multiple comparison post-test when necessary. Beta diversity was quantified using the Bray–Curtis dissimilarity index, and the results were visualized through Principal Coordinates Analysis (PCoA) scatter plots. Differential abundance analyses (Wilcoxon and LEfSe) were conducted on untransformed relative abundance data, which may be influenced by compositional effects. Rarefaction was applied before diversity and differential analyses. The relationship between the Apnea-Hypopnea Index (AHI) and microbiota abundance was examined using simple linear regression, with correlations assessed by Spearman’s rank correlation. The top 20 most abundant microbiota were compared using the Wilcoxon rank-sum test. Linear Discriminant Analysis Effect Size (LEfSe) employed to pinpoint notable taxonomic differences among groups. Differentially represented KEGG pathways were identified using PICRUSt2 analysis. Group-level differences in pathway abundances were evaluated using the Kruskal–Wallis H test. We did not apply NSTI-based filtering, as the primary goal was to detect statistically significant differences in pathway profiles across OSAS subgroups rather than assess per-sample prediction confidence. Due to the exploratory nature of this study and the limited number of tests conducted, *p*-values were reported without multiple-testing correction.

## 3 Results

### 3.1 The gut microbiota features in sICAS patients with OSAS

**Figure 1:**
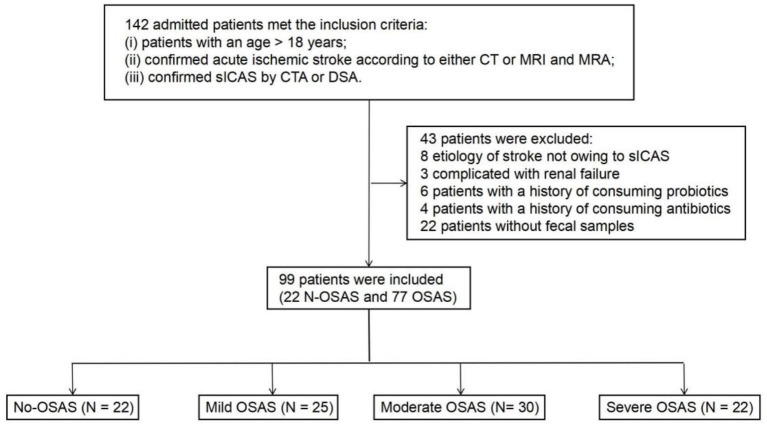
Flow diagram of the patient selection process.

**Figure 2:**
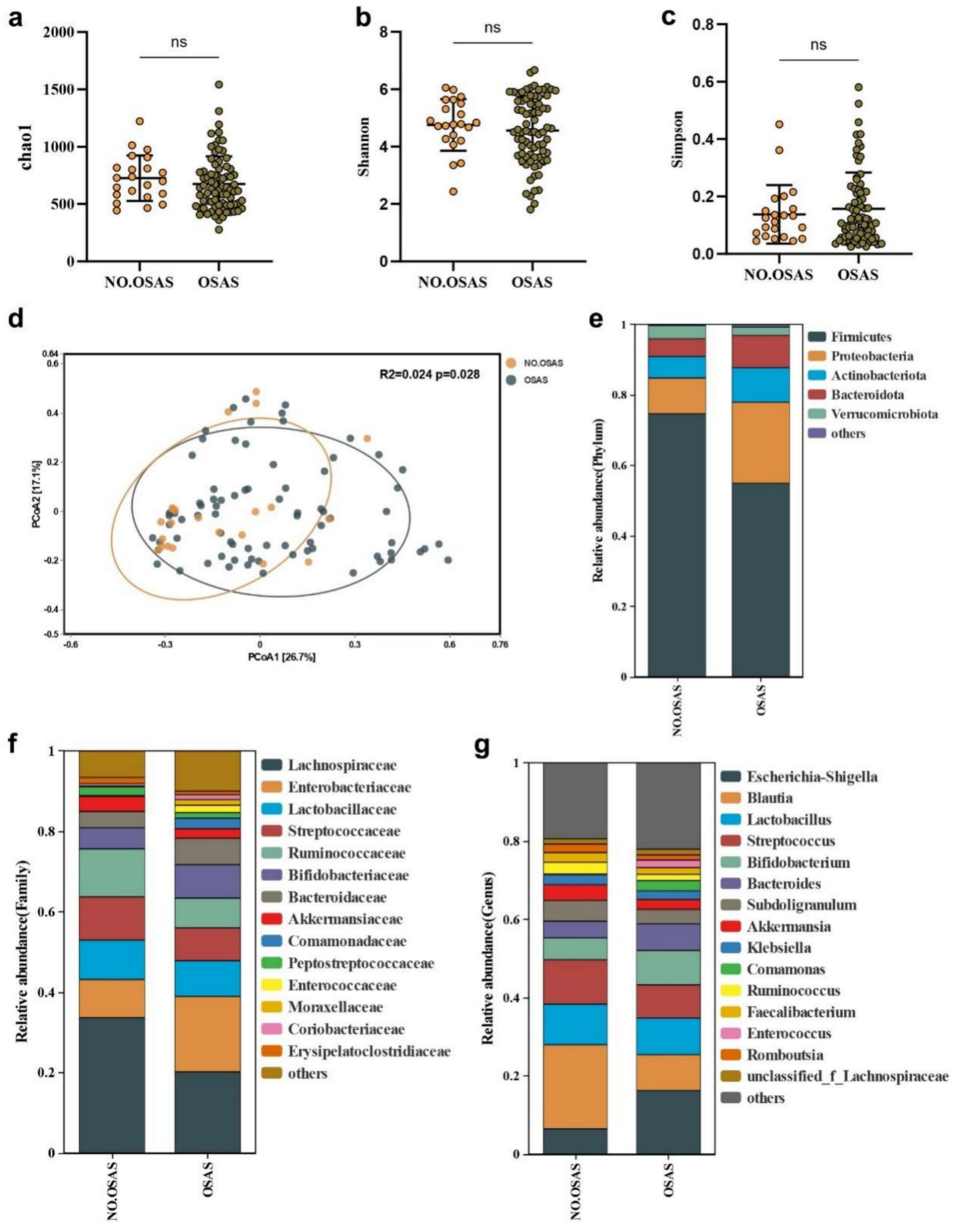
The gut microbiota features in sICAS patients with OSAS. The comparison of *α*-diversity index **(a)** Chao1, **(b)** Shannon index, and **(c)** Simpson index between NO.OSAS and OSAS groups. **(d)** Principal coordinate analysis (PCoA) of the gut microbiota composition based on Bray-Curtis distance. The gut microbiota composition at **(e)** phylum level, **(f)** family level, and **(g)** genus level. Statistical annotations: ns, not significant.

Ninety-nine sICAS patients (mean age 57.6 years, 78% male) were included in the current study (Figure 1; Table 1), including 22 patients without OSAS (NO.OSAS) and 77 patients with OSAS (OSAS), there were no differences in age or gender between the 2 groups (mean age NO.OSAS 57.9 versus OSAS 57.6 years, *p* = 0.90; male NO.OSAS 64% versus OSAS 82%, *p* = 0.07). The OSAS group had a significantly higher AHI and BMI than the NO.OSAS group (25.4 ± 17.7 versus 1.8 ± 1.4, *p* < 0.001, and 23.6 ± 2.1 versus 21.3 ± 2.0, *p* < 0.001, respectively). There were no statistically significant differences in *α*-diversity indexes (Chao1, Shannon, and Simpson) between the OSAS and no-OSAS groups (*p* > 0.05). However, the sample size imbalance (22 vs. 77) may limit the ability to detect subtle differences (Figures 2a–c). The overall gut microbiota compositions were significantly different between the 2 groups based on Bray-Curtis distance (*p* = 0.028) (Figure 2d). At the phylum level, patients with OSAS had a lower abundance of *Firmicutes* and higher abundance of *Proteobacteria* compared to those without OSAS (Figure 2e). At the Class and Order levels, the OSAS group had a decreased abundances of *Clostridia* and *Lachnospirales*, respectively (Supplementary Figures S1a,b). At the Family level, the OSAS group had a decreased abundance of *Lachnospiraceae* (Figure 2f), and at the Genus level, the OSAS group had an increased abundance of *Escherichia-Shigella* and a decreased abundance of *Blautia* (Figure 2g).

**Table 1:** Baseline patient characteristics.

Characteristics	Overall, *N* = 99	NO.OSAS, *N* = 22	OSAS, *N* = 77	*p* value
Demographic				
Age, years	57.6 ± 9.5	57.9 ± 10.1	57.6 ± 9.4	0.90
Male	77 (78)	14 (64)	63 (82)	0.07
Clinical				
AHI	20.2 ± 18.5	1.8 ± 1.4	25.4 ± 17.7	<0.001
DM	28 (28)	5 (23)	23 (30)	0.51
HBP	70 (71)	11 (50)	59 (77)	0.02
Stroke	13 (13)	1 (5)	12 (16)	0.18
CHD	4 (4)	3 (14)	1 (1)	0.01
Smoking	44 (44)	8 (36)	36 (47)	0.39
Drinking	22 (22)	3 (14)	19 (25)	0.27
Metformin	5 (5)	1 (5)	4 (5)	0.90
Statins	7 (7)	2 (9)	5 (6)	0.68
BMI	23.1 ± 2.3	21.3 ± 2.0	23.6 ± 2.1	< 0.001
TG	1.7 ± 1.7	1.5 ± 0.9	1.8 ± 1.9	0.55
TC	4.4 ± 1.3	4.4 ± 1.0	4.4 ± 1.4	0.97
LDL	2.8 ± 1.0	2.8 ± 0.8	2.9 ± 1.0	0.67
HCY	15.4 ± 7.9	17.0 ± 11.3	15.0 ± 6.7	0.33

Data are presented as mean ± standard deviation or *n* (%). AHI, apnea hypopnea index; DM, diabetes mellitus; HBP, high blood pressure; CHD, chronic heart disease; TG, triglyceride; TC, total cholesterol; LDL, low density lipoprotein; HCY, homocysteine. *Data for TG, TC, LDL, and HCY are missing in three subjects.

### 3.2 Comparison of microbiota composition and microbial function between sICAS patients with OSAS and without OSAS

**Figure 3:**
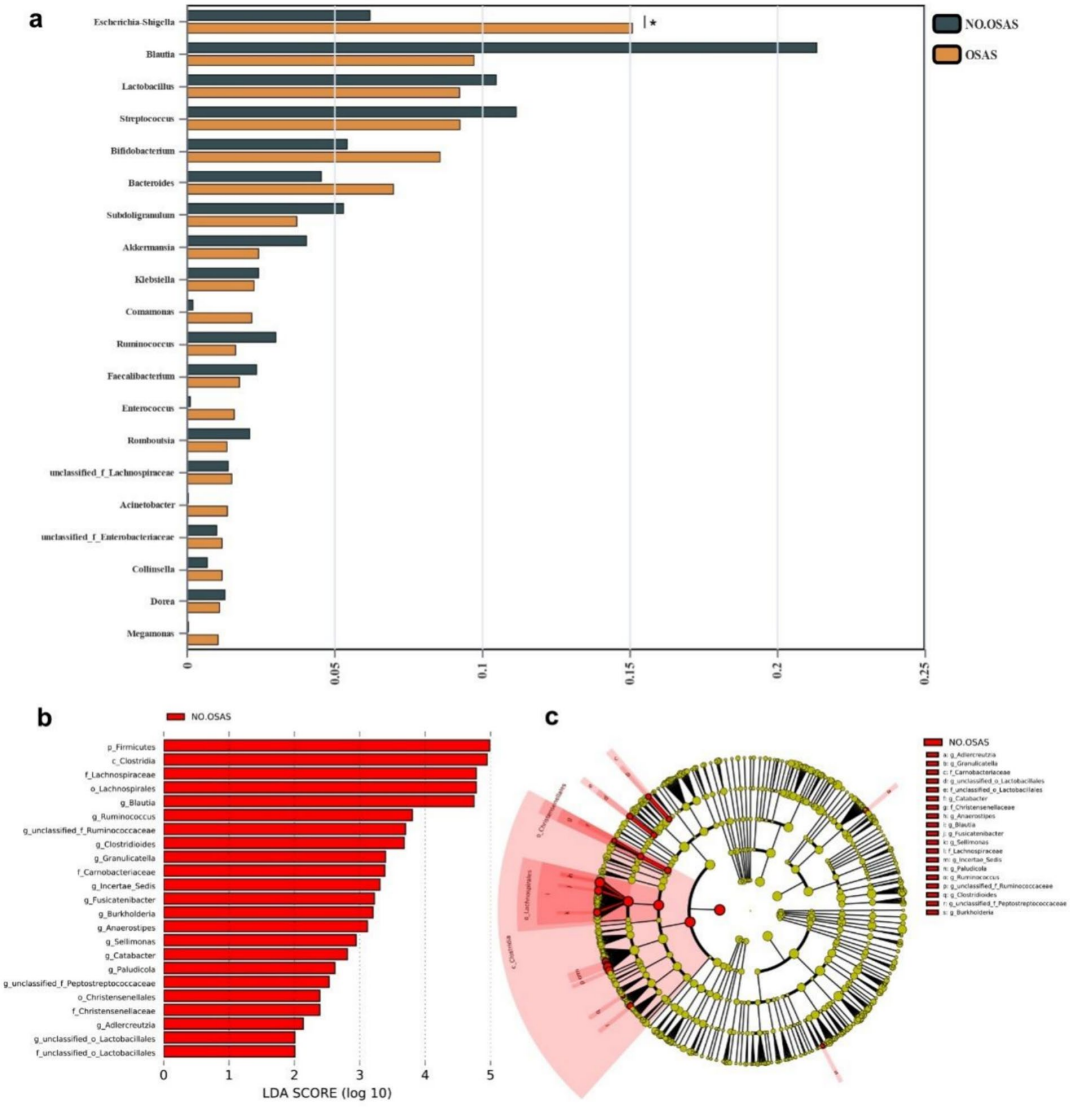
Comparison of microbiota composition between the NO.OSAS and OSAS groups. **(a)** Comparison of the abundances of the top 20 microbiota with the highest relative abundance at the genus level between the 2 groups (Wilcoxon rank-sum test). **(b)** LEfSe showing bacterial taxa with significantly different abundances between the 2 groups (Kruskal–Wallis *H* test, taxa with LDA > 2 were shown). **(c)** Cladogram showing significantly different taxa between the 2 groups. From inner to outer circles, each circle represents a classification level from Phylum to genus. The size of each circle is proportional to its relative abundance. Statistical annotations: *p < 0.05.

**Figure 4:**
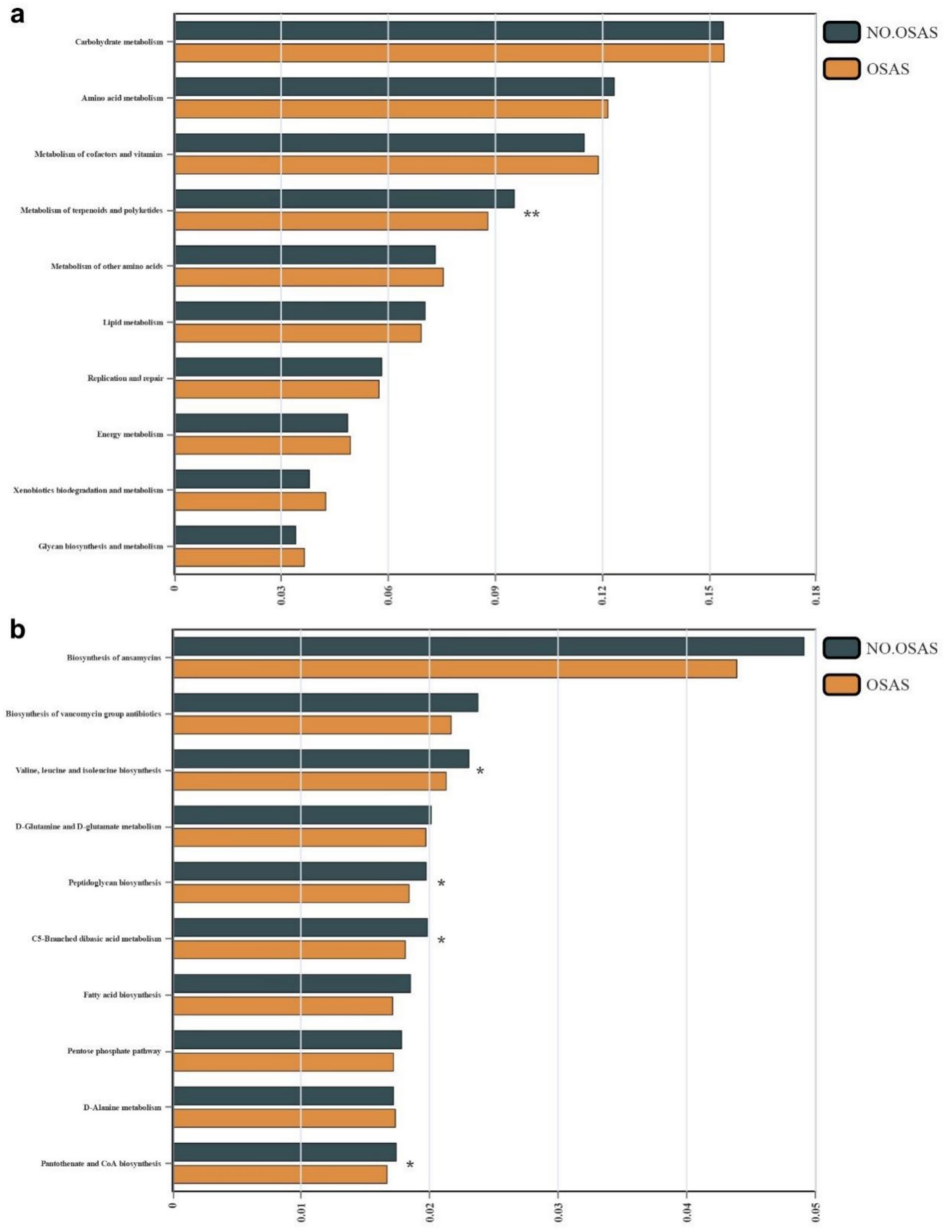
Comparison of microbial KEGG pathways between the NO.OSAS and OSAS groups. **(a)** Comparison of KEGG pathways at level 2 and **(b)** level 3 between the 2 groups. Statistical annotations: *p < 0.05; **p < 0.01. *p* values were computed using the Wilcoxon rank-sum test.

We then compared the abundances of the top 20 microbiota with the highest relative abundance at the Genus level between the 2 groups. We found that the abundance of *Escherichia-Shigella* was significantly increased in the OSAS group (Figure 3a). We further applied a LEfSe analysis across all taxonomy and identified several taxa with significantly increased abundances in the NO.OSAS group, including *Firmicutes*, *Clostridia*, *Lachnospiraceae*, *Blautia*, *Ruminococcus*, etc. (Figures 3b,c). We used PICRUSt analysis to identify differentially present KEGG pathway in gut microbiota. There was no significant differences KEGG categories at Level 1 pathways between the 2 groups (Supplementary Figure S1c), while we identified the metabolism of terpenoids and polyketides at Level 2 pathways predicted to be significantly downregulated in OSAS group (Figure 4a). Additionally, at Level 3 pathways, we found the pathways including Valine, leucine and isoleucine biosynthesis, peptidoglycan biosynthesis, C5-Branched dibasic acid metabolism, and Pantothenate and CoA biosynthesis were significantly downregulated in OSAS group (Figure 4b).

### 3.3 The gut microbiome features in subgroups of OSAS

**Figure 5:**
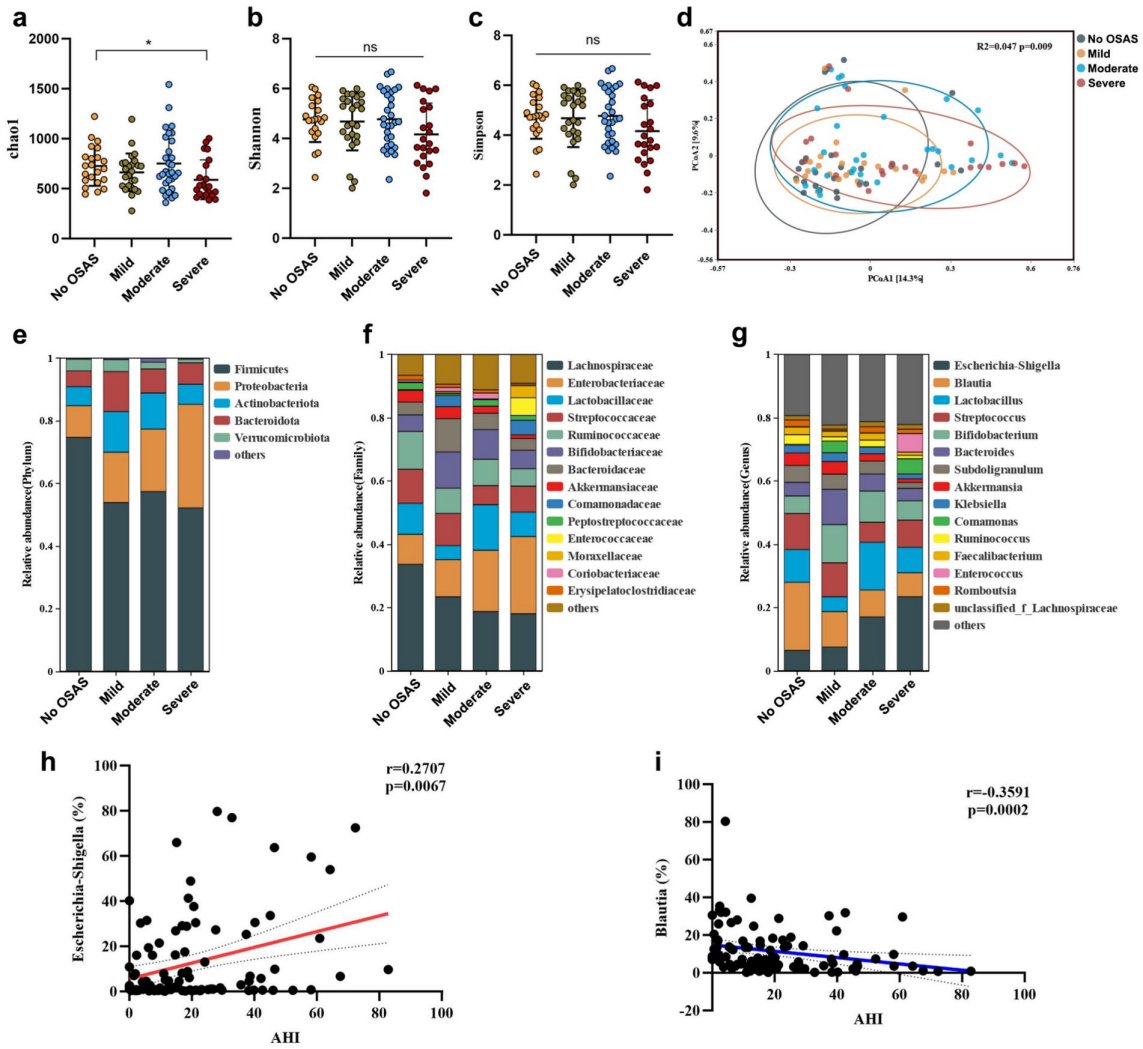
The gut microbiota features in sICAS patients with different severity of OSAS. The α-diversity index **(a)** chao1, **(b)** Shannon index, and **(c)** Simpson index (verall differences were assessed using the Kruskal–Wallis test followed by Dunn’s multiple-comparison post-test when appropriate). **(d)** Principal coordinate analysis (PCoA) of the gut microbiota composition based on Bray-curtis distance. The gut microbiota composition at **(e)** phylum level, **(f)** family level, and **(g)** genus level. Spearman’s correlations between AHI and the abundances of **(h)** *Eschericha-Shigella* and **(i)** *Blautia*. Statistical annotations: ns, not significant; *p < 0.05.

The OSAS group was subdivided based on the severity of OSAS. The severity of OSAS was defined by the apnea-hypopnea index (AHI). AHI < 5 was considered no sleep apnea (OSAS.0), AHI 5–15 was defined as mild OSAS, AHI 15–30 as moderate OSAS, and AHI > 30 as severe OSAS. There were no significant differences in age or sex between the 4 groups (Table 2). Although the Shannon and Simpson indexes were comparable between the 4 group, the chao1 index of OSAS.3 group was significantly reduced compared to that of OSAS.0 group (Figures 5a–c). The gut microbiota compositions were significantly different between the 4 groups, showing an increasing distance from OSAS.0 to OSAS.3 (Figure 5d). At the phylum level, the abundance of *Proteobacteria* tended to increase with increasing OSAS severity (Figure 5e). At the Class level, the abundance of *Clostridia* tended to decrease while the abundance of *Gammaprotebacteria* tended to increase with increasing OSAS severity (Supplementary Figure S2a). At the Order level, the abundances of Lachnospirales and Oscillospirales tended to decrease with increasing OSAS severity (Supplementary Figure S2b). At the Family level, the abundance of *Lachonospiraceae* tended to decrease while the abundance of *Enterobacteriaceae* tended to increase with increasing OSAS severity (Figure 5f). At the Genus level, the abundance of *Escherichia-Shigella* tended to increase while the abundance of *Blautia* tended to decrease with increasing OSAS severity (Figure 5g). Notably, the abundance of *Escherichia-Shigella* was positively associated with AHI (Figure 5h) while the abundance of *Blautia* displayed negative association with AHI (Figure 5i).

**Table 2:** Baseline patient characteristics, stratified by AHI value.

Characteristics	No-OSAS, *N* = 22	Mild, *N* = 25	Moderate, *N* = 30	Severe, *N* = 22	*p* value
Demographic					
Age, years	57.9 ± 10.1	57.9 ± 7.5	59.1 ± 9.4	55.1 ± 11.1	0.53
Male	14 (64)	17 (68)	26 (87)	20 (91)	0.06
Clinical					
AHI	1.8 ± 1.4	10.0 ± 3.0	20.8 ± 4.5	49.4 ± 13.4	<0.001
DM	5 (23)	6 (24)	10 (33)	7 (32)	0.82
HBP	11 (50)	21 (84)	21 (70)	17 (77)	0.07
Stroke	1 (5)	5 (20)	6 (20)	1 (5)	0.18
CHD	3 (14)	1 (4)	0 (0)	0 (0)	0.053
Smoking	8 (36)	9 (36)	16 (53)	11 (50)	0.49
Drinking	3 (14)	8 (32)	7 (23)	4 (18)	0.48
Metformin	1 (5)	2 (8)	1 (3)	1 (5)	0.88
Statins	2 (9)	2 (8)	2 (7)	1 (5)	0.94
BMI	21.3 ± 2.0	22.7 ± 1.8	23.1 ± 2.0	25.1 ± 1.8	<0.001
TG	1.5 ± 0.9	1.9 ± 1.4	1.8 ± 1.0	1.7 ± 1.7	0.93
TC	1.0 ± 0.2	1.1 ± 0.2	1.7 ± 0.3	1.1 ± 0.2	0.15
LDL	2.8 ± 0.8	3.2 ± 0.9	2.5 ± 1.0	3.0 ± 0.9	0.04
HCY	16.9 ± 11.3	13.7 ± 3.5	15.2 ± 9.8	16.0 ± 3.8	0.57

Data are presented as mean ± standard deviation or *n* (%). AHI, apnea hypopnea index; DM, diabetes mellitus; HBP, high blood pressure; CHD, chronic heart disease; TG, triglyceride; TC, total cholesterol; LDL, low density lipoprotein; HCY, homocysteine. *Data for TG, TC, LDL, and HCY are missing in three subjects.

### 3.4 Comparison of microbiota composition and microbial function between subgroups of OSAS

**Figure 6:**
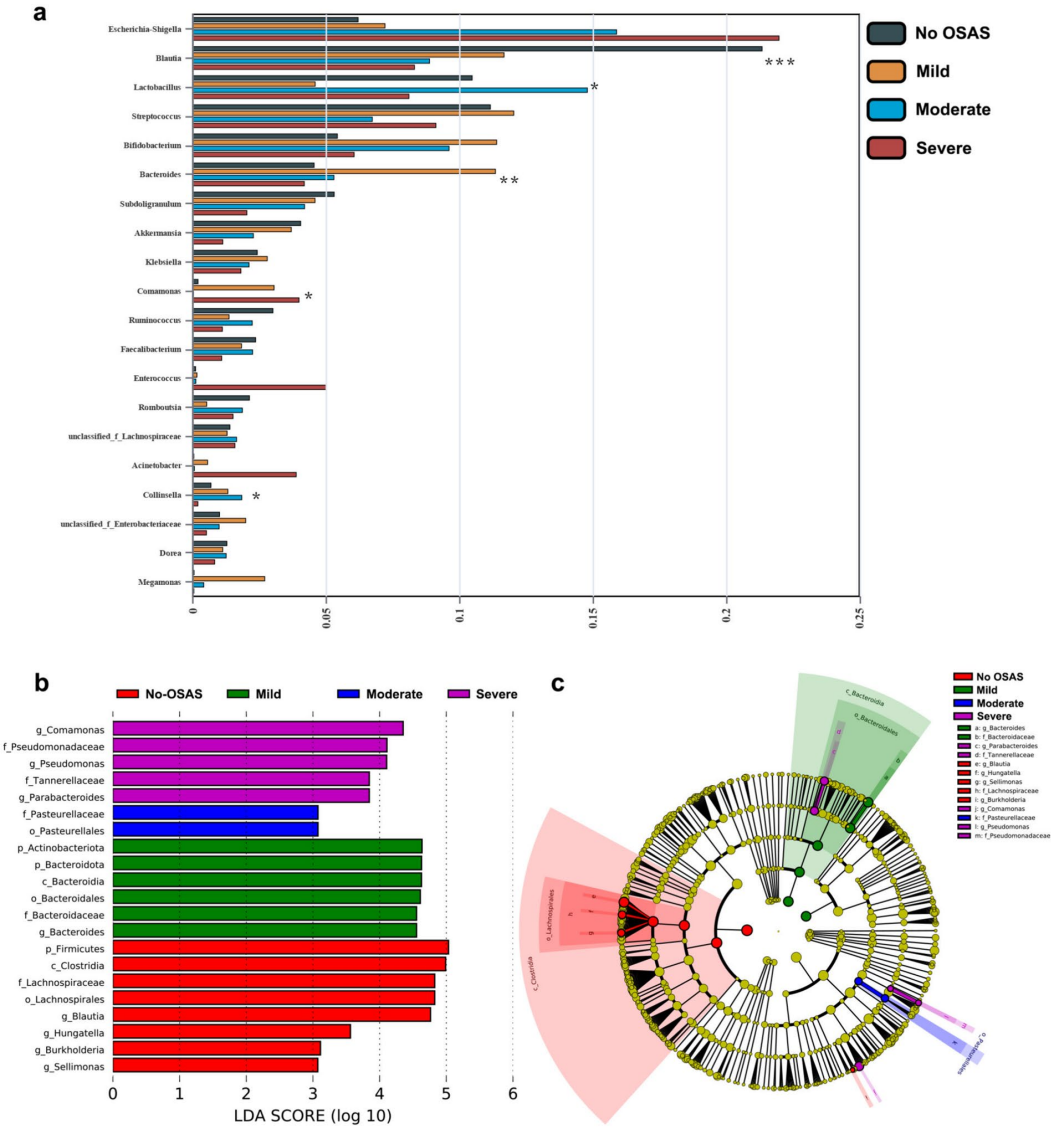
Comparison of microbiota composition in sICAS patients with different severity of OSAS. **(a)** Comparison of the abundances of the top 20 microbiota with the highest relative abundance at the genus level between the 4 groups (overall differences were assessed using the Kruskal–Wallis test followed by Dunn’s multiple-comparison post-test when appropriate. Asterisks placed next to individual taxa/pathways indicate that the corresponding feature differs significantly between groups). **(b)** LEfSe showing bacterial taxa with significantly different abundances between the 4 groups (Kruskal–Wallis H test, taxa with LDA > 3 were shown). **(c)** Cladogram showing significantly different taxa between the 4 groups. From inner to outer circles, each circle represents a classification level from Phylum to genus. The size of each circle is proportional to its relative abundance. Statistical annotations: *p < 0.05; **p < 0.01; ***p < 0.001.

**Figure 7:**
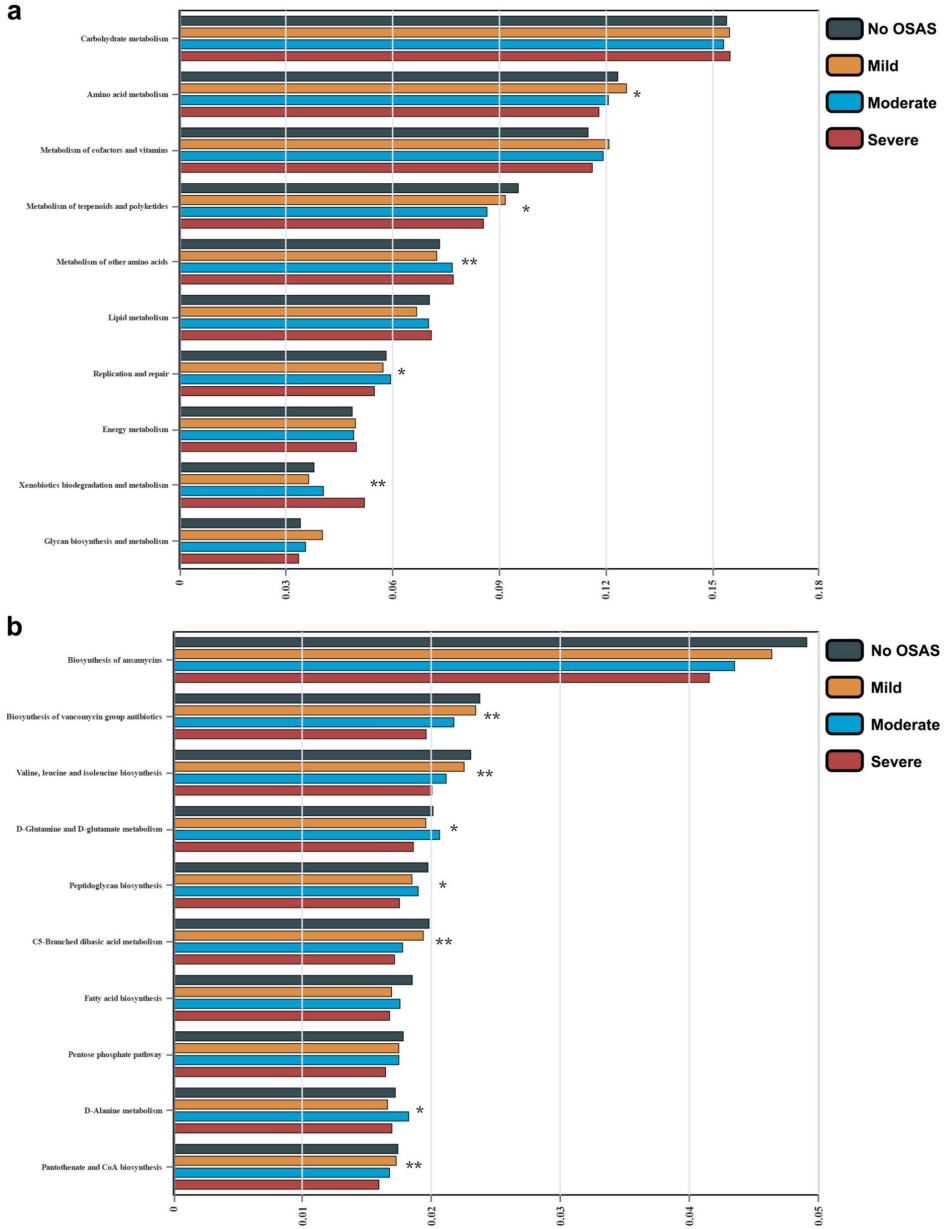
Comparison of microbial KEGG pathways in sICAS patients with different severity of OSAS. **(a)** Comparison of KEGG pathways at Level 2 and **(b)** Level 3 between the 4 groups. Statistical annotations: *p < 0.05; **p < 0.01. Overall differences were assessed using the Kruskal–Wallis test followed by Dunn’s multiple-comparison post-test when appropriate. Asterisks placed next to individual taxa/pathways indicate that the corresponding feature differs significantly between groups under the statistical test specified above.

We compared the abundances of the top 20 microbiota with the highest relative abundance at the Genus level and found the abundances of *Blautia*, *Lactobacillus*, *Bacteroides*, *Comamonas*, and *Collinsella* were significantly different between the 4 groups (Figure 6a). Moreover, LEfSe analysis revealed consistently depleted abundances of *Firmicutes*, *Clostridia*, *Lachnospiraceae*, and *Blautia* in OSAS patients with OSAS (Figures 6b,c). PICRUSt analysis revealed Genetic information processing at Level 1 KEGG pathway to be significantly different pathway between the 4 groups (Supplementary Figure S2c). At Level 2 KEGG pathways, Amino acid metabolism, Metabolism of terpenoids and polyketides, Metabolism of other amino acids, Replication and repair, Xenobiotics biodegradation and metabolism were significantly different pathways between the 4 groups (Figure 7a). At Level 3 KEGG pathways, Biosynthesis of vancomycin group antibiotics, Valine, leucine and isoleucine biosynthesis, D-Glutamine and D-glutamate metabolism, Peptidoglycan biosynthesis, C5-Branched dibasic acid metabolism, D-Alanine metabolism, and Pantothenate and CoA biosynthesis were significantly different pathways between the 4 groups (Figure 7b).

## 4 Discussion

To date, the research on the crosstalk between sleep disturbance and gut microbiota dysbiosis is at its infancy (Neroni et al., 2021). Whether there is an associative or causal relationship between OSAS and gut microbiota in sICAS patients remains unknown. Since OSAS is a risk factor for atherosclerosis (most probably through inflammatory process) (McNicholas, 2009; Ciccone and Scicchitano, 2012; Ji et al., 2019), and It has been noted that the gut microbiota is a key factor in the progression of atherosclerosis (Witkowski et al., 2020), whether OSAS is associated with alterations in the gut microbiota that co-occur with atherosclerosis remains an interesting topic. In the current study, we showed that patients with OSAS did exhibit a dysbiotic gut microbiota, featuring in increased abundance of pathogenic bacteria most notably *Escherichia-Shigella* and decreased abundances of several beneficial bacteria including *Blautia*, *Lachnospiraceae*, and *Runminococcus*. More importantly, with severer OSAS (higher AHI), the abundance of *Escherichia-Shigella* tends to increase and the abundances of those beneficial microbes tend to decrease.

A key feature of this study is that all participants had sICAS. We selected this population for two reasons. First, sICAS represents a clinically high-risk cerebrovascular phenotype in which modifiable comorbidities such as OSAS may meaningfully influence outcomes; thus, understanding OSAS-associated microbiome patterns is particularly relevant in this setting. Second, restricting enrollment to sICAS reduces etiological heterogeneity in cerebrovascular disease and enables a more focused evaluation of whether OSAS severity stratifies gut microbial community structure within a relatively homogeneous vascular phenotype. Importantly, because our cohort did not include non-ICAS controls, the present data do not establish microbiome signatures attributable to ICAS itself; rather, they characterize microbiome differences associated with OSAS severity within an sICAS population.

At the phylum level, the increase in the abundance of *Proteobacteria* has been proposed as a microbial signature of gut microbiota dysbiosis (Shin et al., 2015), some researchers have suggested *Proteobacteria* to be a common factor in human diseases (Rizzatti et al., 2017). Indeed, patients with metabolic disorders or inflammation and cancer tend to have higher proportion of *Proteobacteria* than healthy subjects (Shin et al., 2015). Interestingly, transplantation of *Enterobacter cloacae* B29 (a bacteria strain belonging to *Proteobacteria*), which was isolated from the gut microbiota of obese human, to germ-free mice, was capable of inducing insulin resistance and obesity in recipient mice (Fei and Zhao, 2013). Another animal study has also revealed that a *Proteobacteria*-dominated gut microbiota structure predisposes the host to chronic colitis in a genetically susceptible mouse model (Carvalho et al., 2012). Notably, in human study exploring the association between the gut microbiota and ischemic stroke, it has been reported that patients with severer brain injury have higher abundance of *Enterobacteriaceae* (a microbiota at the Family level belonging to *Proteobacteria*), which is also an independent risk factor for primary unfavorable outcome of stroke (Xu et al., 2021). Additionally, many studies have revealed an important role of *Enterobacteriaceae* in stroke injury and prognosis (Wang et al., 2022; Wang et al., 2021). As such, it is possible that OSAS may be associated with gut microbiota dysbiosis in sICAS patients, although causality cannot be inferred. At the Genus level, we found the severity of OSAS was positively associated with the abundance of *Escherichia-Shigella*. It has been found that the expansion of *Escherichia-Shigella* in the intestine is linked to the activation Nod-like receptors protein 3 (NLRP3) inflammasome (Li et al., 2020). Interesting, a diet high in processed meat and low in vegetable leads to an enrichment of gut *Escherichia-Shigella*, which can be reversed by a diversified diet (Zhang et al., 2020). As such, the entangling relationships between diet, gut microbiota, and OSAS are worthy of future investigation.

The depletion of beneficial bacteria, such as *Blautia*, *Lachnospiraceae*, and R*unminococcus*, was also a prominent feature of gut dysbiosis in OSAS patients. *Lachnospiraceae*, *Blautia*, and R*unminococcus* are known as the SCFA-producing bacteria, serving as the major contributor to the production of butyrate (Louis and Flint, 2017). Butyrate has been widely acknowledged to possess anti-inflammatory and immunomodulating abilities in the host. It represents the major source of nutrient and energy for epithelial cells in the colon and is important in the maintenance of the intestinal barrier by regulating the expression of the tight junction protein and epithelial growth (Rivière et al., 2016). Furthermore, butyrate has been found to participate in the maintenance of the blood–brain barrier (Braniste et al., 2014), and protection against metabolic diseases (Sanna et al., 2019). The enrichment of *Escherichia-Shigella* in OSAS patients may promote systemic inflammation through activation of innate immune pathways, particularly the NLRP3 inflammasome, which has been implicated in both gut barrier dysfunction and cerebrovascular injury. In contrast, *Blautia* is a key SCFA-producing genus known to support gut barrier integrity, suppress pro-inflammatory cytokine production, and improve host metabolic profiles. The depletion of *Blautia* may thus reduce the availability of protective SCFAs such as butyrate, potentially exacerbating systemic inflammation and vascular injury pathways involved in stroke pathogenesis. In our study, the abundance of *Blautia* was negatively associated with AHI, suggesting a protective role of this microbiota in OSAS. Indeed, *Blautia* has been of particular interest by researchers as a potential probiotic with its contribution to ameliorating inflammation and metabolic disorders and its antibacterial activity against pathogens (Liu et al., 2021). Although we observed notable alterations in the relative abundance of *Blautia* and *Escherichia-Shigella* in OSAS patients with sICAS, we did not measure circulating markers such as LPS, TMAO, SCFAs, IL-6, or CRP that would provide direct evidence of systemic inflammation or metabolic impact. This limits our ability to directly link microbial shifts to clinical outcomes, and future studies should include such measurements to evaluate the functional relevance of gut dysbiosis in this population. Moreover, while the term “dysbiosis” is commonly used to describe microbial imbalances, its interpretation remains context-dependent. The magnitude of fold changes observed in our study, particularly the enrichment of *Escherichia-Shigella* and depletion of SCFA-producing *Blautia* is comparable to microbial shifts reported in patients with inflammatory bowel disease, metabolic syndrome, or post-stroke conditions. Nevertheless, standardized benchmarks for defining clinically meaningful dysbiosis remain an area of ongoing research.

Importantly, these OSAS-associated microbial shifts were observed in a cohort in which all participants had symptomatic intracranial atherosclerotic stenosis, suggesting that OSAS may act as a modifier of the gut ecosystem in a high vascular-risk population. The pattern we observed that enrichment of potentially pro-inflammatory taxa (e.g., *Proteobacteria*/*Enterobacteriaceae*-related genera such as *Escherichia-Shigella*) together with depletion of putative SCFA-producing *Firmicutes* (e.g., *Lachnospiraceae*/*Blautia*/*Ruminococcus*-related taxa), is consistent with a dysbiosis profile that could plausibly amplify atherosclerosis-related processes via impaired gut-barrier function, increased endotoxin burden, and heightened systemic inflammation and endothelial dysfunction (Salazar et al., 2023). In parallel, microbiome-derived metabolites implicated in atherogenesis (e.g., TMAO) and protective SCFAs (e.g., butyrate) provide additional mechanistic candidates linking OSAS-associated dysbiosis to vascular disease phenotypes (Zhu et al., 2020). Although we did not measure circulating LPS/TMAO/SCFAs in this study, recent work in intracranial atherosclerotic stenosis has reported concurrent alterations in the gut microbiome and systemic metabolites, supporting the plausibility of a gut-vascular axis in ICAS (Zhang et al., 2024). Collectively, these considerations help unify our findings by positioning OSAS-associated dysbiosis as a potential biological intermediate that may exacerbate vascular inflammation and metabolic risk in sICAS.

There were several KEGG pathways that were significantly different between OSAS patients and those without OSAS, and among different severities of OSAS. While PICRUSt2 does provide NSTI values to estimate the phylogenetic distance between ASVs and reference genomes, we did not filter pathways based on NSTI scores. This decision was based on our focus on group-level comparisons rather than individual prediction confidence. However, we acknowledge that NSTI reporting can improve transparency and may be considered in future analyses. Also, although functional predictions suggest alterations in microbial metabolic pathways such as butyrate biosynthesis, we did not perform wet-lab validation using qPCR or targeted metabolomics in this study. Future studies should incorporate such validations to confirm key functional shifts and enhance the biological relevance of predictive findings.

Our study has several important limitations. First, we did not collect dietary data such as food frequency questionnaires or 24-h recalls. Since diet is a primary driver of gut microbiota composition, especially affecting taxa like *Escherichia-Shigella* and SCFA-producing bacteria, this unmeasured variable may confound our findings. Second, we did not assess bowel movement patterns or Bristol Stool scores, which may also influence microbiome structure and correlate with OSAS severity. Moreover, while we recorded use of certain medications such as metformin, and statins, these variables were not incorporated into multivariable statistical models. Future studies with larger sample sizes and more comprehensive phenotyping are needed to validate our results and disentangle these complex interactions. We acknowledge that all differential abundance analyses in our study were conducted on relative abundance data without applying centered log-ratio (CLR) or alternative compositional data transformations. As microbiome data are inherently compositional, this approach may introduce spurious statistical inferences due to the constant-sum constraint. Advanced methods such as CLR transformation, ALDEx2, or ANCOM-BC are better suited for such analyses and should be incorporated in future studies. Additionally, we did not generate volcano plots showing CLR-based effect sizes and adjusted *p*-values, which may aid in the interpretability of microbial shifts. These are valuable suggestions for follow-up analyses. Although we applied rarefaction in our diversity analyses, we did not repeat the differential testing using cumulative sum scaling (CSS) or GMPR normalization. Lastly, polysomnography was performed after the onset of acute stroke, and post-stroke sleep architecture changes may have transiently influenced AHI measurements. The severity of OSAS was positively associated with BMI, which itself is a major determinant of gut microbiota composition; although this trend was observed, we were unable to fully adjust for BMI due to sample size limitations. The study cohort was predominantly male (78%), had a relatively small sample size, and lacked a non-ICAS comparator group. These factors may limit generalizability and introduce potential sex-related microbiome bias. Future studies with larger, sex-balanced cohorts and appropriate control groups are warranted.

It is important to acknowledge that the cross-sectional design of this study limits causal inferences. The observed associations between OSAS and gut microbiota alterations do not imply directionality. It is plausible that a pre-existing dysbiotic gut microbiome, or unmeasured confounding variables such as dietary patterns, medication use (e.g., proton pump inhibitors), bowel habits, or intermittent hypoxia-induced inflammation, may contribute to both OSAS severity and stroke risk. Longitudinal or interventional studies are required to disentangle these complex relationships.

The observed microbiome alterations in OSAS patients raise the question of whether these changes are reversible. Continuous positive airway pressure (CPAP) therapy, the standard treatment for OSAS, has been reported to partially restore gut microbial balance and reduce inflammation in small-scale studies. In parallel, the depletion of SCFA-producing genera such as *Blautia* suggests that targeted probiotic supplementation could be a viable strategy to improve gut barrier function and systemic inflammation. Future interventional studies should explore whether such therapies can effectively reshape the microbiome and influence stroke outcomes in sICAS patients.

This study has several strengths. First, we examined gut microbiome profiles in a clinically well-characterized cohort with sICAS and systematically stratified participants by OSAS severity, enabling evaluation of microbiome differences across a clinically meaningful spectrum. Second, fecal sampling and DNA extraction followed a standardized workflow, and we combined community-level analyses with differential taxa identification and predictive functional profiling to provide a multi-level view of dysbiosis. Nonetheless, to more fully delineate the role of microbiome disruption in OSAS and its vascular relevance, future studies should (i) include appropriate comparator groups (e.g., non-ICAS OSAS, non-OSAS controls, and ideally healthy controls), (ii) adopt longitudinal designs and evaluate the impact of OSAS therapy (e.g., CPAP) on microbiome trajectories, and (iii) incorporate multi-omics and host profiling, such as shotgun metagenomics, metatranscriptomics, fecal and serum metabolomics (SCFAs, bile acids, TMAO), and circulating markers of inflammation, gut barrier integrity, and endothelial function, to validate inferred functions and clarify causal pathways.

In conclusion, we found that sICAS patients with OSAS did exhibit a prominent dysbiosis of the gut microbiota, characterized by enriched pathogenic bacteria such as *Escherichia-Shigella* and depleted beneficial bacteria such as *Blautia*, whose abundance were significantly associated with OSAS severity. The microbial metabolic pathway of peptidoglycan biosynthesis was found to be downregulated in patients with severe OSAS, suggesting a potentially altered microbial functional profile that warrants validation in future mechanistic and multi-omics studies. Future studies incorporating inflammatory and metabolic biomarkers will be essential to confirm the systemic relevance of the observed microbiome alterations.

## Data Availability

The original contributions presented in the study are included in the article/Supplementary material, further inquiries can be directed to the corresponding author.
