# Supplementary material for: Impact of obstructive sleep apnea on gut microbiome of patients with symptomatic intracranial atherosclerotic stenosis
Source: Front Aging Neurosci. 2026 Jan 22;18:1713733. doi: 10.3389/fnagi.2026.1713733 (PMC12872767; doi:10.3389/fnagi.2026.1713733)
Supplement: Supplementary file 1 [file Data_Sheet_1.docx]

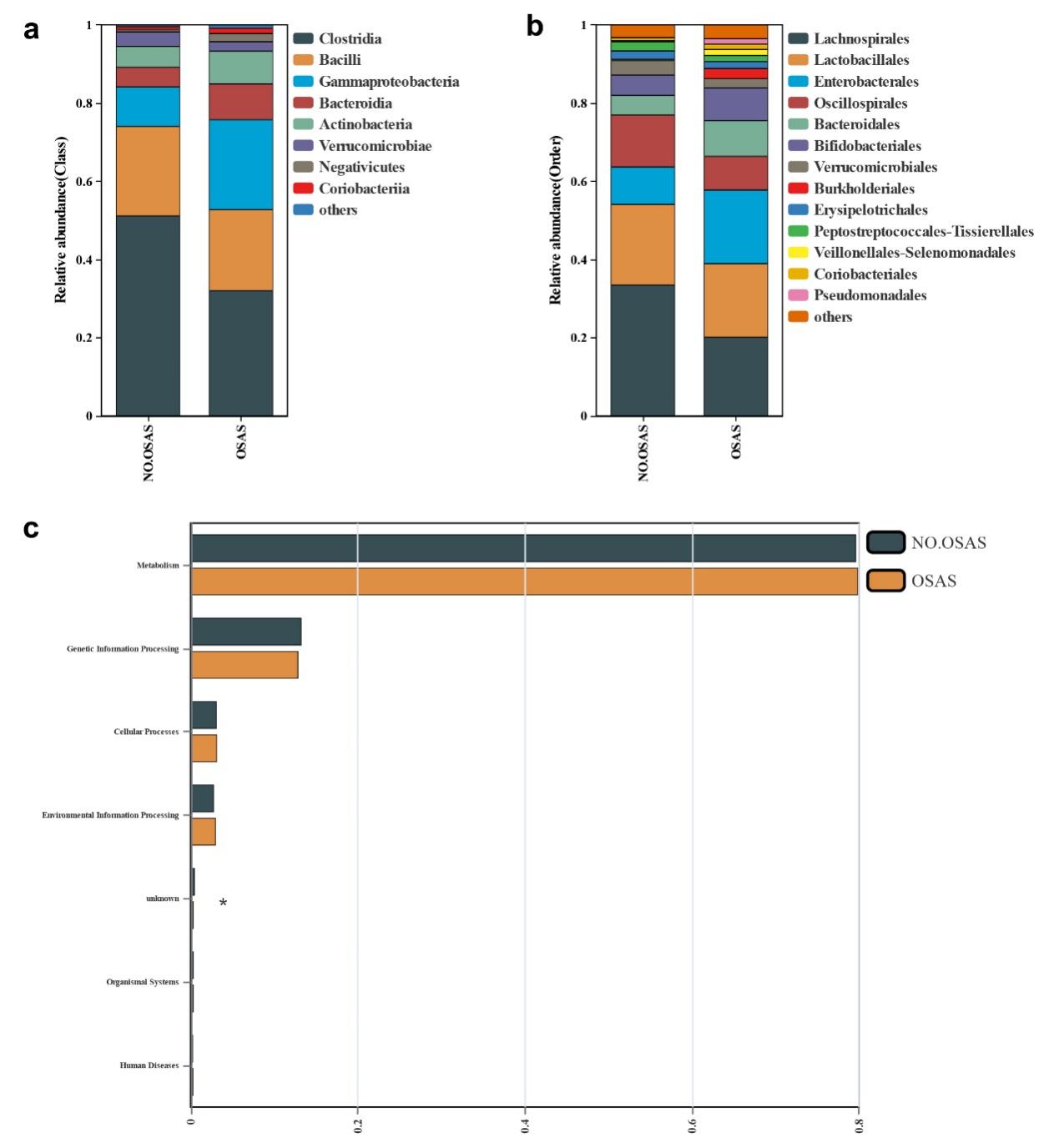


**Figure S1.** The gut microbiota features and microbial KEGG pathways in sICAS patients with OSAS. The gut microbiota composition at (a) Class level, (b) Order level. (c) Comparison of KEGG pathways at Level 1 between the 2 groups.





**Figure S2.** The gut microbiota features and microbial KEGG pathways in sICAS patients with different severity of OSAS. The gut microbiota composition at (a) Class level, (b) Order level. (c) Comparison of KEGG pathways at Level 1 between the 4 groups.
